# The Unique and Joint Associations of Childhood and Recent Life Stressors on Chronic Pain Symptoms

**DOI:** 10.3390/jcm15186968

**Published:** 2026-09-09

**Authors:** Scott G. Ravyts, Emily C. Rosado, Michael Persin

**Affiliations:** Department of Psychological Science, The University of North Carolina at Charlotte, 9105 University Rd., Charlotte, NC 28223, USA; erosado3@charlotte.edu (E.C.R.); persin1@charlotte.edu (M.P.)

**Keywords:** trauma, adversity, chronic pain

## Abstract

**Background/Objectives:** Early life stress is a well-established predictor of chronic pain; however, less is known about whether early and recent life stressors interact to influence chronic pain. The purpose of the present study was to examine the unique and joint associations of early and recent life stressors on chronic pain symptoms and examine whether general sensory sensitivity mediates the association between stress exposure and pain-related symptoms. **Methods**: Participants included 577 adults from the Multidisciplinary Approach to the Study of Chronic Pelvic Pain (MAPP-II) Symptoms Pattern Study. Early life stressors (ELSs) and recent life stressors (RLSs) were examined using the Childhood and Recent Traumatic Events Scale, while general sensory sensitivity and pain symptoms were measured using the Complex Medical Symptoms Inventory and the Brief Pain Inventory. **Results:** The interaction between ELSs and RLSs significantly predicted pain severity (*β* = 0.25, *p* = 0.01) and distribution (*β* = 0.25, *p* = 0.02); these results remained significant even after controlling for sociodemographic characteristics. By contrast, ELSs (*β* = 0.15, *p* = 0.04) and RLSs (*β* = 0.16, *p* = 0.02) uniquely predicted pain interference. Moderated mediation models indicated that RLSs moderated the effect of ELSs on general sensory sensitivity, but there was no evidence of an indirect association with pain-related outcomes. **Conclusions:** Consistent with the early life sensitization model of stress, the interaction between ELSs and RLSs is associated with worse pain-related symptoms. Additional research is needed to better understand the mechanisms through which stress exposure influences chronic pain.

## 1. Introduction

Individuals with chronic pain experience disproportionally high rates of severe, chronic stress [1]. Not only is chronic stress a risk factor for the onset of chronic pain but it is also associated with worse clinical outcomes, including greater pain severity, worse pain interference, and more widespread pain [2,3,4]. Despite the consequences of lifetime stress exposure on pain, child and adult stressors have predominately been examined separately. Little is known about whether these stressors have unique and independent effects on pain or whether the interplay between childhood and adult stressors has a particularly negative effect. Relatedly, the biobehavioral mechanisms connecting stress exposure to chronic pain are not well understood.

### 1.1. Theoretical Models of Stress and Health Outcomes

Stress has been defined as a situation in which environmental demands surpass one’s natural regulatory abilities, particularly in situations characterized by unpredictability and uncontrollability [5]. While adverse childhood events have long been known to predict chronic pain [6], more recent research suggests that stressors in adulthood, especially if they are severe or repeated, are linked with poor pain outcomes [7,8]. Additional research is needed to understand how stress becomes biologically embedded and contributes to adverse health outcomes. Several competing theories have been put forth. For example, the stress generation model, originally developed to explain depression and later expanded to other health conditions, states that individuals exposed to early life stressors are more likely to experience greater stressors in adulthood, thereby contributing to adverse health outcomes [9]. By contrast, the stress accumulation model posits that stress exposure has a cumulative effect over time and that it is the accumulation of stress which leads to poor health consequences [10,11]. Finally, the early life-sensitization model posits that early life stressors independently predict health-related consequences and amplify the responses to subsequent stressors in adulthood, thereby contributing to poor health outcomes [12]. Although these competing theories about the health-related effects of stress and adversity have been applied to a range of chronic health conditions, less is known about how the timing and frequency of stressful life events specifically relates to chronic pain. Understanding which model of stress best predicts clinical pain outcomes may inform assessment and intervention efforts for chronic pain, especially among individuals with nociplastic pain conditions for whom stress and trauma may be a significant risk factor leading to central nervous system dysfunction [13].

### 1.2. Mechanisms Linking Stressful Life Events and Pain

Additional research is also needed to better understand the mechanisms connecting stressful life events to chronic pain. Neurobiological mechanisms such as general sensory sensitivity have been identified as one potential mechanism, with preliminary research suggesting that trauma exposure is linked with heightened sensory sensitivity across several chronic pain populations [14,15]. General sensory sensitivity may be a particularly salient mechanism, as trauma can increase vigilance to sensory information and promote heightened threat detection [16], leading individuals to notice and react more strongly to bodily sensations. Stress exposure may also contribute to central sensitization, resulting in amplified processing of sensory input across multiple domains, including pain [17]. Despite growing interest in the role of general sensitivity in pain-related symptoms, it is unknown whether the timing and accumulation of stressful life events is linked with general sensory sensitivity and its pain-related consequences.

### 1.3. Present Study

The purpose of the present study is to use the aforementioned existing theoretical frameworks of stress and health to: (1) examine the unique and joint associations between early and recent life stressors and chronic pain symptoms (i.e., pain severity, interference, and distribution), and (2) determine whether general sensory sensitivity mediates the association between stressful life events and pain-related symptoms. In order to answer these research questions, baseline data from the second phase of the Multidisciplinary Approach to the Study of Chronic Pelvic Pain (MAPP II) were used.

## 2. Methods

### 2.1. Participants

The MAPP-II Symptoms Pattern Study (SPS) is the second phase of an observational study designed to examine current and prospective outcomes relating to men and women with urologic chronic pelvic pain [18]. Participants for MAPP II SPS were recruited from 2015 to 2019 from six sites in the United States. Men and women were included if they were 18 years or older, reported pelvic pain or discomfort present for the majority of the past 3 months, and had received a clinical diagnosis of interstitial cystitis/bladder pain syndrome (IC/BPS) or chronic prostatitis/chronic pelvic pain syndrome (CP/CPPS). Full inclusion and exclusion criteria are described elsewhere [18]. Only baseline self-reported data were used for the current secondary data analysis. The MAPP-II study protocol was approved by the institutional review board of each of the six participating study sites and participants provided informed consent prior to participating in the study. Additional informed consent was not required for this secondary analysis of existing, de-identified, data.

### 2.2. Measures

**Pain Symptoms:** The Brief Pain Inventory (BPI) was used to measure pain severity, interference, and distribution [19]. Participants were asked to rate their current pain as well as their average, least, and worst pain over the last week on an 11-point Likert scale from 0 (*No Pain*) to 10 (*Pain as bad as you can imagine*). A pain severity score was calculated by averaging the four responses. Cronbach’s alpha for the BPI pain severity subscale was 0.89, which is consistent with good internal consistency. Pain interference was assessed by asking participants to rate how much their pain has interfered with various life domains (e.g., work, mood, sleep) from 0 (*Does not interfere*) to 10 (*Completely interferes*). Pain interference was calculated by averaging the seven items. Cronbach’s alpha for the BPI pain interference subscale was 0.94, corresponding to excellent internal consistency. Finally, the distribution of participants’ pain was calculated via question number two on the BPI. Areas where participants reported experiencing any pain greater than zero were summed to estimate the distribution of a participant’s pain (ranging from 0 to 76).

**Early Life Stressors:** The presence of traumatic events before the age of 17 was assessed by the Childhood Traumatic Events Scale (CTES) [20]. The CTES includes six items which assess childhood exposure to stressful or traumatic events (e.g., death of a family member, traumatic sexual experience) on a yes or no scale. Scores on the CTES range from 0 to 6, with each positively endorsed item resulting in one point.

**Recent Life Stressors:** Exposure to recent traumatic events was assessed using the Recent Traumatic Events Scale (RTES) [20]. The scale assesses participants’ exposure to seven discrete, potentially traumatic life events (e.g., serious accident, physical or sexual assault, natural disaster, or life-threatening illness) occurring within the past three years. Participants indicated whether they have experienced each event, with items coded dichotomously to reflect event occurrence. A cumulative trauma score was calculated by summing endorsed events, with scores ranging from 0 to 7 and higher scores indicating greater exposure to recent life stressors.

**General Sensory Sensitivity:** Consistent with prior research [21], items from the Complex Medical Symptoms Inventory were used to assess general sensory sensitivity [22]. Specifically, a general sensory sensitivity index was derived from 4 items inquiring about chemical sensitivity, sensitivity to sounds, sensitivity to odors, and sensitivity to bright lights. For each item, participants indicated whether they experienced the symptom for at least 3 months during the past year. Cronbach’s alpha for this scale was 0.85, indicating high internal consistency. Items were summed to generate a total score ranging from 0 to 4, with higher scores reflecting greater sensory sensitivity. If one or more of the four items was missing, a total score was not calculated.

**Anxiety and Depression:** Anxiety and depressive symptoms were assessed using the Hospital Anxiety and Depression Scale (HADS) [23]. The HADS is a 14-item self-report measure designed to assess current anxiety and depressive symptomology while minimizing the influence of somatic complaints. The scale consists of two 7-item subscales, Anxiety (HADS-A) and Depression (HADS-D). Items are rated on a 4-point Likert scale yielding subscale scores ranging from 0 to 21, with higher scores indicating greater symptom severity. Cronbach’s alpha for the anxiety and depression subscales was 0.83 and 0.87, respectively. The HADS has demonstrated good internal consistency, construct validity, and sensitivity across clinical and non-clinical populations.

### 2.3. Data Analysis

Only participants who completed the BPI at baseline were included in the current analyses. Multiple linear regression models were used to examine the independent and interactive effect of early life stressors (ELSs) and recent life stressors (RLSs) on pain intensity, interference, and distribution. ELSs and RLSs were not mean-centered prior to calculating an interaction term, as a score of zero for these variables represents the absence of trauma exposure and was considered a meaningful and interpretable reference point. Both unadjusted models and models controlling for sociodemographic characteristics (i.e., age, sex, race, ethnicity, and education) were conducted. Positive associations between ELSs and pain, coupled with a non-significant association between ELSs and pain after controlling for RLSs or sociodemographic covariates, were interpreted as supporting the stress generation model. Main effects for ELSs and RLSs without a significant interaction term were considered to support the stress accumulation model. Finally, a significant interaction term, indicating that individuals with high levels of ELSs and RLS exhibit the greatest level of pain, was considered as evidence for the early life-sensitization model.

The association between stressful life events and pain severity or interference via general sensory sensitivity was examined using PROCESS Macro Version 4.0 Model 7 [24]. Specifically, moderated mediation models using 5000 bootstrapped resamples and a 95% confidence interval were used in which ELS was entered as the predictor variable, general sensory sensitivity was entered as the mediator variable and RLSs were included as a moderator between ELSs and general sensory sensitivity. Pain severity or interference was included as the respective criterion variable for each moderated mediation model. Sociodemographic characteristics including age, sex, African American race, ethnicity, and education were included as covariates. Missing data were handled using complete-case analysis and only included participants with complete data for all variables included in the model.

## 3. Results

### 3.1. Participant Characteristics

The sample consisted of 577 adults with urologic chronic pelvic pain syndrome (mean age = 44.75; *SD* = 15.67). Participants were predominantly female (66.6%), White (91.7%), and non-Hispanic (93.5%). Rates of interpersonal violence in childhood were high, with 22.6% of participants reporting a history of childhood sexual trauma and 18.1% reporting a history of childhood physical abuse. Rates of recent interpersonal violence were lower with 4.2% reporting recent sexual abuse and 5.0% reporting sexual or physical violence within the last three years. The correlation between ELSs and RLSs was 0.33 (*p* < 0.001). A cross-tabulation frequency table of ELSs by RLSs is presented in the Supplemental Materials. A fourth of participants (25.1%) were above the cut-off for anxiety while 16.1% of participants endorsed clinically significant symptoms of depression. Finally, a tenth of the sample (10.3%) reported having been diagnosed with PTSD. Complete demographic and clinical information is presented in Table 1.

### 3.2. Multiple Linear Regressions

**Pain Severity:** ELSs were associated with greater pain severity (*β* = 0.24, *p* < 0.001). When RLSs and the interaction between RLSs and ELSs were entered into the model, only the interaction term was significant (*β* = 0.23, *p* = 0.03), indicating that the association between ELSs and pain severity was stronger for individuals with more RLSs. This interaction term remained significant after controlling for sociodemographic characteristics (*β* = 0.25, *p* = 0.01). The complete results of these multiple linear regressions are presented in Table 2.

**Pain Interference:** The results of multiple regression analyses indicated that ELSs (*β* = 0.15, *p* = 0.04) and RLSs (*β* = 0.16, *p* = 0.02) were each uniquely associated with greater pain interference. The bivariate association between ELSs and pain interference (*β* = 0.28, *p* < 0.001) decreased when RLSs were entered into the model but remained significant while the interaction between ELSs and RLSs was not significant (*β* = 0.10, *p* = 0.31). Finally, the unique associations between ELSs and RLSs were no longer significant when controlling for sociodemographic characteristics. The results of these linear regressions are presented in Table 3.

**Pain Distribution:** ELSs (*β* = 0.23, *p* < 0.001) predicted the number of pain sites; however, when RLS and the interaction between ELS and RLS were entered into the model, only the interaction was significant (*β* = 0.25, *p* = 0.02), indicating that the association between ELS and pain distribution was stronger for individuals with more RLS. When controlling for sociodemographic characteristics, the interaction term (*β* = 0.26, *p* = 0.01) remained significant. These multiple linear regressions are presented in Table 4. A graphical representation of the significant interaction terms for pain severity and distribution is presented in the Supplemental Materials.

### 3.3. Mediational Models

**Pain Severity:** When controlling for sociodemographic variables, a moderated mediation model (*n* = 557) indicated that RLSs moderated the association between ELSs and general sensory sensitivity (*R*^2^ = 0.01, *p* = 0.04) and that general sensory sensitivity independently predicted greater pain intensity (*B* = 0.19, *p* < 0.001). However, the indirect effect of ELSs on pain severity via general sensory sensitivity was not significant (*B* = 0.03, 95% CI: −0.01, 0.08), as was the index of moderated mediation (Index = −0.01, 95% CI: −0.02, 0.01).

**Figure 1:**
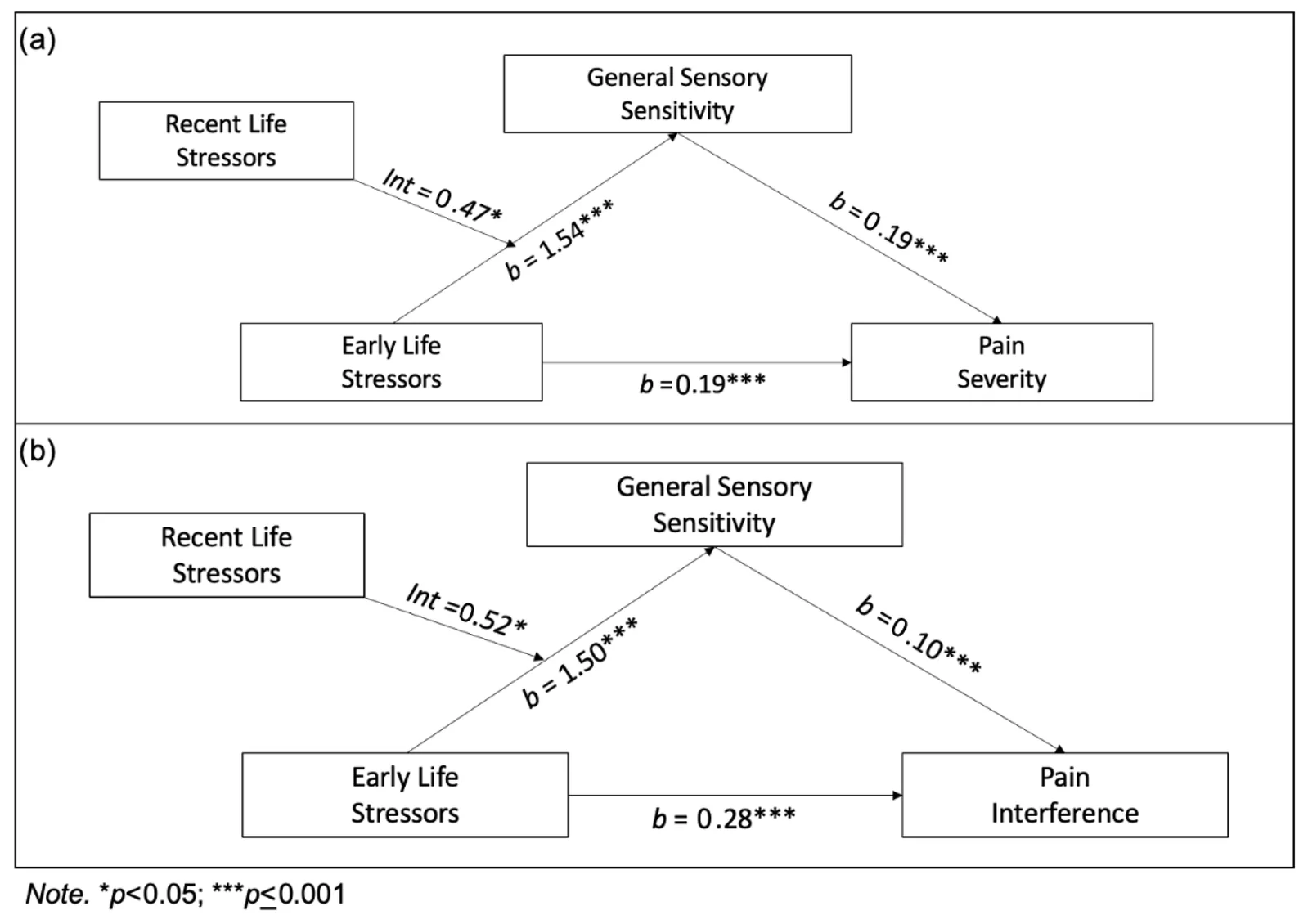
Moderated mediation model of life stress, general sensory sensitivity, and pain outcomes for (**a**) pain severity and (**b**) pain interference.

**Pain Interference:** Replicating this moderated mediation model with pain interference (*n* = 561) as the outcome produced similar results. Specifically, RLSs moderated the relationship between ELSs and general sensory sensitivity (*R*^2^ = 0.01, *p* = 0.03), while general sensory sensitivity directly predicted pain interference (*B* = 0.10, *p* < 0.001). By contrast, there was no indirect effect of ELS on pain interference via general sensory sensitivity (*B* = 0.05, 95% CI: −0.02, 0.13) and the index of moderated mediation was non-significant (Index = −0.01, 95% CI: −0.05, 0.02). Moderated mediation models for pain severity and interference are presented in Figure 1.

## 4. Discussion

Despite growing interest in the role of stress and trauma exposure in chronic pain, there is a lack of research regarding whether ELSs and RLSs combine or interact to predict chronic pain. In order to address this gap, we examined the unique and joint associations of ELSs and RLSs with pain severity, interference, and distribution among adults with chronic pelvic pain, as well as whether these associations could be partially explained by increased general sensory sensitivity. We found that the interaction between ELSs and RLSs was associated with pain severity and distribution beyond either type of stressor alone. By contrast, general sensory sensitivity did not mediate the association between stress exposure and various pain-related outcomes.

Our findings regarding the interplay between ELSs and RLSs is consistent with the early life sensitization model whereby early childhood stress exposure may be associated with heightened sensitivity to subsequent stress; however, the present observational data cannot establish a biological sensitization mechanism. These patterns of findings are largely consistent with previous research examining other health outcomes. For example, in a large cohort study of older adults, greater daily stress exposure was associated with more self-reported chronic health conditions; however, this relationship was heightened among adults with more exposure to adverse childhood events [25]. Similarly, in another study comprising over thirty thousand adults, childhood adversity moderated the relationship between adult life stress and various forms of psychopathology [26]. Collectively, these outcomes suggest that greater attention should be given to more proximal stressors, particularly among adults with known histories of early childhood adversity.

Our finding that ELSs and RLSs interacted to predict pain severity and distribution, but not pain interference, suggests that early life sensitization may be primarily associated with the sensory experience of pain rather than its functional consequences. Pain interference is determined by a broader set of factors, including psychological functioning and social context. Consequently, while exposure to stress across the lifespan may be associated with increased pain, additional psychological and social factors may modify the degree to which stress translates into functional impairment. For example, both social support and meaning making are believed to blunt the impact of trauma exposure and chronic pain [27,28]. Further research is needed to examine whether the associations between ELSs, RLSs, and chronic pain outcomes are replicated among adults with other types of chronic pain conditions, especially among conditions associated with high rates of both trauma exposure and functional impairment such as fibromyalgia [29].

Contrary to our hypothesis, general sensory sensitivity did not mediate the association between stress exposure and either pain severity or interference. Nevertheless, general sensory sensitivity independently was significantly associated with both pain severity and interference. Although some research suggests that general sensory sensitivity predicts chronic pain prospectively [30,31], additional research examining the temporal relationship between general sensory sensitivity and pain is needed. It is possible that general sensory sensitivity and chronic pain develop concurrently through shared underlying processes. While ELS was associated with both general sensory sensitivity and pain-related outcomes, future research should also investigate alternative mechanisms through which lifetime stress exposure contributes to chronic pain. For example, stress exposure may alter stress physiology (e.g., HPA axis and autonomic system dysregulation), increase inflammation, or contribute to greater emotion dysregulation which may, in turn, contribute to worse pain outcomes. Existing theoretical models of chronic pain and trauma exposure, such as the mutual maintenance model or shared vulnerability model [32], have primarily focused on psychosocial mechanisms while biological mechanisms connecting trauma to chronic pain remain understudied.

### Implications

The current findings suggest that taking a developmental approach to the assessment of trauma exposure may be beneficial in the treatment of chronic pain. While approximately one-fifth of participants reported a history of childhood physical or sexual abuse, recent life stressors were also common, with 85.9% of participants reporting experiencing at least one significant life stressor in the past three years, and 35.3% reporting three or more recent stressors. Although there have been more calls for clinicians to implement trauma-informed care and screen for the presence of adverse childhood events [33], assessment of recent life stressors and its pain-related consequences has largely been overlooked. Rather than relying primarily on retrospective inventories of childhood adversity, clinicians may benefit from inquiring about trauma across the lifespan including a more detailed evaluation of recent and ongoing stress, as well as using trauma exposure inventories which include questions about trauma exposure in adulthood.

Adopting a developmental approach to clinical assessment may also inform treatment planning, as individuals with high trauma exposure may be good candidates for treatments that directly address these life experiences and their emotional consequences. For example, newer psychological interventions for nociplastic pain, such as Emotional Awareness and Expression Therapy (EAET), directly acknowledge the role of life stressors by bringing a patient’s awareness to the relationship between the timing of these stressors and the onset of one’s physical symptoms [34]. Recent research among individuals with chronic musculoskeletal conditions suggests that trauma-informed psychological interventions such as EAET may outperform cognitive behavioral therapy for chronic pain and lead to greater reductions in pain severity and emotional distress [35]. Additional research is needed to better understand whether trauma-informed interventions may be beneficial to adults with chronic pelvic pain.

## 5. Limitations

The current findings should be interpreted within the context of several limitations. First, the current findings are cross-sectional; thus, causal conclusions cannot be established. Additional longitudinal research is needed to untangle the associations between life stressors, general sensory sensitivity, and chronic pain symptoms. Secondly, the assessment of ELS and RLS was limited and subject to recall bias; future research should seek to replicate these findings using more robust measures of trauma and stress exposure which include the frequency, perceived severity, chronicity, and developmental timing of various types of stressful life events. Finally, the current sample was predominantly composed of White females. Additional research is needed to better understand whether the current findings generalize to more racially diverse patient populations who are more likely to be exposed structural racism and experience race-based traumatic events which have been linked to disparities in pain-related outcomes [36]. Similarly, future research should replicate the current findings among individuals with other chronic pain conditions, including chronic pain populations with high rates of trauma exposure (e.g., fibromyalgia). Despite these limitations, the study had several strengths, including a large sample size and theory-driven framework that allowed us to better understand the impact of life stressors on chronic pain.

## 6. Conclusions

Our preliminary findings indicate that associations between RLSs and pain-related symptoms are worse for individuals with more ELSs; this pattern of findings is consistent with the early-life sensitization model of stress. Given the high rates of trauma exposure among adults with chronic pain, additional research is needed to better understand the mechanisms through which lifetime stress exposure contributes to chronic pain symptoms and how psychological interventions for pain can best be harnessed to address these factors.

## Figures and Tables

**Table 1:** Demographic and clinical characteristics.

	Mean (SD) or %	Range
Age	44.75 (15.67)	18–79
Gender		
Male	33.40%	--
Female	66.60%	--
Race		
White	91.70%	--
African American	6.30%	--
Asian	1.90%	--
Native American	2.30%	--
Native Hawaiian	0.30%	--
Other	2.80%	--
% Hispanic	6.50%	--
BPI-Pain Severity	3.77 (2.01)	0–9
BPI-Pain Interference	3.46 (2.61)	0–10
BPI-Pain Distribution	7.06 (8.33)	0–76
General Sensory Sensitivity	1.16 (1.48)	0–4
HADS-Anxiety	7.34 (4.78)	0–21
HADS-Depression	5.77 (4.55)	0–20

*Note.* BPI = Brief Pain Inventory; Hospital Anxiety and Depression Scale. Respondents could select more than one race.

**Table 2:** Multiple linear regression predicting pain severity.

Model		B	Std. Error	Beta	t	Sig.
1	Constant	3.16	0.13			
	ELS	0.36	0.06	0.24	5.75	<0.001
2	Constant	3.21	0.21		15.19	<0.001
	ELS	0.08	0.11	0.06	0.71	0.48
	RLS	0.02	0.11	0.01	0.15	0.88
	ELS × RLS	0.10	0.04	0.23	2.19	0.03
3	Constant	3.62	0.75		4.82	<0.001
	ELS	0.04	0.11	0.04	0.39	0.70
	RLS	−0.06	0.11	−0.04	−0.52	0.61
	ELS × RLS	0.11	0.04	0.25	2.48	0.01
	Age	−0.01	0.01	−0.08	−1.88	0.06
	Sex (1 = female)	0.19	0.18	0.04	1.03	0.30
	Hispanic	−0.14	0.33	−0.02	−0.42	0.67
	African American	1.30	0.34	0.16	3.89	<0.001
	Education	0.01	0.00	0.08	1.92	0.06

*Note.* ELS = Early Life Stressors; RLS = Recent Life Stressors; Model 1: *F*(1, 556) = 33.01, *p* < 0.01, *R*^2^ = 0.06; Model 2: *F*(3, 554) = 15.61, *p* < 0.01, *R*^2^ = 0.08; Model 3: *F*(8, 549) = 9.30, *p* < 0.01, *R*^2^ = 0.12.

**Table 3:** Multiple linear regression predicting pain interference.

Model		B	Std. Error	Beta	t	Sig.
1	Constant	2.54	0.17		15.22	<0.001
	ELS	0.50	0.07	0.28	6.97	<0.001
2	Constant	2.17	0.27		8.14	<0.001
	ELS	0.27	0.13	0.15	2.01	0.04
	RLS	0.32	0.14	0.16	2.35	0.02
	ELS × RLS	0.06	0.06	0.10	1.02	0.31
3	Constant	2.04	0.95		2.15	0.03
	ELS	0.23	0.13	0.13	1.75	0.08
	RLS	0.24	0.14	0.12	1.76	0.08
	ELS × RLS	0.07	0.06	0.13	1.27	0.21
	Age	−0.01	0.01	−0.07	−1.75	0.08
	Sex (1 = female)	0.22	0.23	0.04	0.97	0.33
	Hispanic	0.13	0.41	0.01	0.31	0.76
	African American	1.39	0.42	0.13	3.28	0.00
	Education	0.01	0.00	0.09	2.25	0.03

*Note*. ELS = Early Life Stressors; RLS = Recent Life Stressors; Model 1: *F*(1, 560) = 48.61, *p* < 0.01, *R*^2^ = 0.08; Model 2: *F*(3, 558) = 25.40, *p* < 0.01, *R*^2^ = 0.12; Model 3: *F*(8, 553) = 12.44, *p* < 0.01, *R*^2^ = 0.15.

**Table 4:** Multiple linear regression predicting pain distribution.

Model		B	Std. Error	Beta	t	Sig.
1	Constant	4.69	0.54		8.66	<0.001
	ELS	1.31	0.23	0.23	5.60	<0.001
2	Constant	5.06	0.87		5.79	<0.001
	ELS	0.22	0.44	0.04	0.51	0.61
	RLS	−0.03	0.45	0.00	−0.06	0.95
	ELS × RLS	0.43	0.18	0.25	2.37	0.02
3	Constant	0.65	3.16		0.21	0.84
	ELS	0.06	0.44	0.01	0.13	0.90
	RLS	−0.22	0.46	−0.03	−0.49	0.63
	ELS × RLS	0.46	0.18	0.26	2.55	0.01
	Age	0.00	0.02	0.00	0.09	0.93
	Sex (1 = female)	2.49	0.75	0.14	3.32	<0.001
	Hispanic	0.32	1.40	0.01	0.23	0.82
	African American	−1.13	1.38	−0.03	−0.82	0.41
	Education	0.01	0.01	0.04	0.92	0.36

*Note.* ELSs = Early Life Stressors; RLSs = Recent Life Stressors. Model 1: *F*(1, 556) = 31.33, *p* < 0.01, *R*^2^ = 0.05; Model 2: *F*(3, 554) = 15.15, *p* < 0.01, *R*^2^ = 0.08; Model 3: *F*(8, 549) = 7.04, *p* < 0.01, *R*^2^ = 0.10.

## Data Availability

Data from MAPP-II (https://doi.org/10.58020/2gk3-hy95 (accessed on 1 May 2025)) reported here are available for request via the NIDDK Central Repository (NIDDK-CR), Resources for Research (R4R) at https://repository.niddk.nih.gov (accessed on 1 May 2025).

## Supplementary Materials

The following supporting information can be downloaded at: https://www.mdpi.com/article/10.3390/jcm15186968/s1, Table S1: Crosstabulation of Recent Life Stressors by Early Life Stressors; Figure S1: Interaction Between Early Life Stress and Recent life Stress on Pain Severity and Distribution.
