# Supplementary material for: The Unique and Joint Associations of Childhood and Recent Life Stressors on Chronic Pain Symptoms
Source: J Clin Med. 2026 Sep 9;15(18):6968. doi: 10.3390/jcm15186968 (PMC13607032; doi:10.3390/jcm15186968)
Supplement: Supplementary file 1 [file jcm-15-06968-s001.zip › jcm-4505320-supplementary.pdf]

Supplemental Table S1. Crosstabulation of Recent Life Stressors by Early Life Stressors.

		RLS								
	Count	0	1	2	3	4	5	6	7	Total
ELS	0	32	44	24	16	4	0	0	0	120
	1	27	66	36	19	8	3	0	0	159
	2	13	33	42	27	5	2	0	1	123
	3	5	23	31	14	11	0	1	0	85
	4	3	12	11	13	10	2	1	0	52
	5	0	5	5	10	3	0	1	0	24
	6	0	1	1	3	0	1	0	0	6
Total		80	184	150	102	41	8	3	1	569

Note. ELS = Early Life Stressors; RLS = Recent Life Stressors

Supplemental Figure S1. Interaction Between Early Life Stress and Recent life Stress on Pain Severity and Distribution.

a) Pain Severity

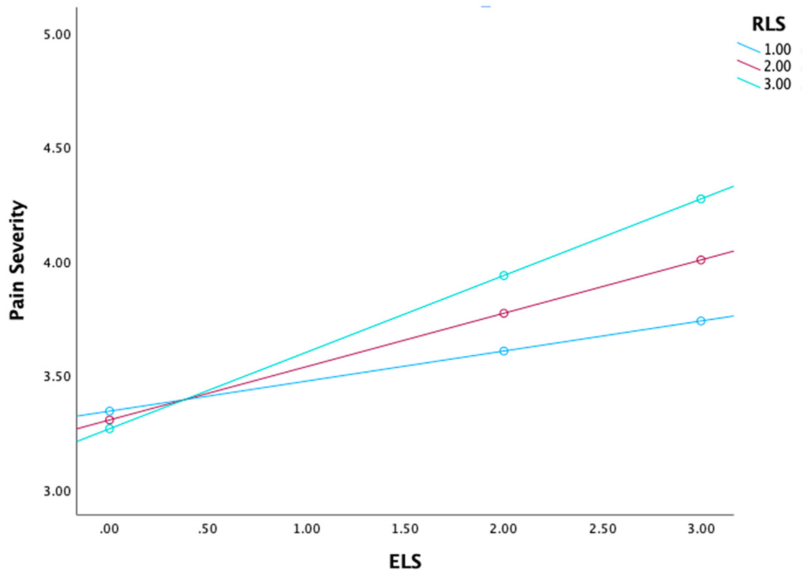

b) Pain Distribution

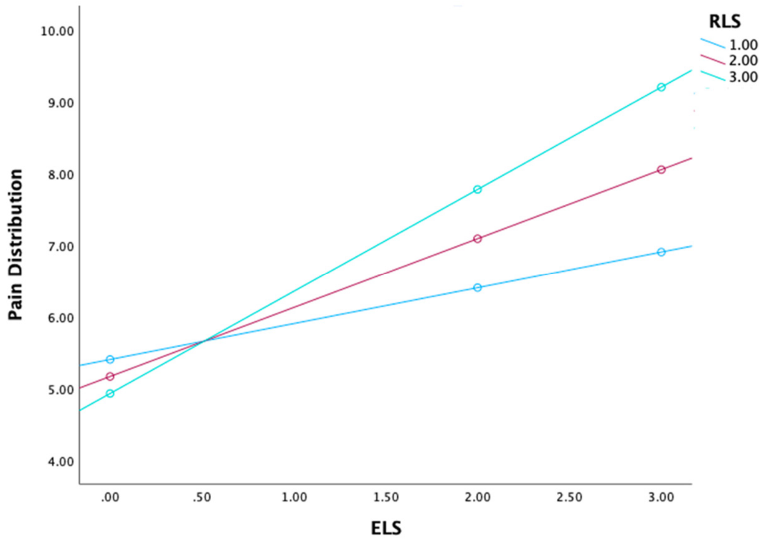

Note. RLS = Low (16th percentile), Average (50th percentile), and High (84th percentile) levels of Recent Life Stress. ELS = Early Life Stress.
